# Gastrostomy

**Published:** 1894-12-22

**Authors:** 


### Gastrostomy.

The surgery of the stomach has advanced of late years in a truly remarkable manner. Not only for diseases of that organ itself, but also for conditions affecting the œsophagus, the surgeon is called upon to practise his art for the relief of suffering and the prolongation of life. The course of an organic stricture of the œsophagus when left to itself, and even under palliative treatment, is at best but a miserable existence. The stenosis goes on increasing until the patient, unable to be fed even by the catheter, has to resort to nutrient enemata, and after a time even this means of sustenance fails, and the patient dies of starvation. It was in order, if possible, to obviate such a miserable death that the operation of gastrostomy was instituted. Originally proposed by Egeberg, a Norwegian military surgeon, in 1837, Sédillot gave the operation its name and indications in 1846, and performed the first gastrostomy on the human subject at Strasbourg in 1849. The results of operation were, however, for many years far from satisfactory. The surgeon did not usually see the patient until the last possible moment, when even fluids could not pass the œsophagus, and the patient was *in extremis*. The great trouble seems to have been the oozing out and regurgitation of the food through and at the side of the tube in the gastric fistula. Thus not enough was retained to nourish the patient, and the oozing caused a troublesome eczema in the epigastric region. Happily there are now several methods at the surgeon's disposal which will enable him to prevent regurgitation from a gastric fistula, so that the patient need not now die of starvation. From an admirable sketch of the present position of this operation (*Am. Journ. Med. Sci.*, Oct., 1894) by Meyer, we find that the old method—the "method of Fenger"—was the only one in use for forty years, but the ill-success attending it caused surgical interference to fall into disrepute. It is gratifying to note, therefore, that no less than four methods have recently been introduced, which tend to overcome the difficulties formerly experienced, and promise much better results.

By one or other of these devices the ordinary course of organic stricture of the œsophagus may be shorn of much of its misery, and there can be no doubt that, in view of what can now be done for them, these cases should be referred to the surgeon in the early, instead of in the last, stage of the disease.

Mikulicz says, "As soon as fluids and semi-solid food find some resistance in passing down," and, according to Meyer, "as soon as the scales show a steady, though perhaps slow, decrease in the patient's weight," gastrostomy is indicated. By the time the patient has reached a state of inanition he can ill bear an operation, and it may be too late for the stomach again to resume its normal function. Gastrostomy should not be a last resort. Let the surgeon see these cases early. that he may be able to select his operation according to the character of the stenosis. An early operation will be less dangerous and will be much more likely to prolong life in a case that will eventually require gastrostomy, for, as we have seen, the surgeon can at any rate prevent the fearful termination of death by starvation. After colotomy for cancer of the rectum there is often marked improvement in the patients' general condition and the malignant growth gives less trouble. The same holds good for gastrostomy for carcinoma of the œsophagus, and even in cases of burn of the œsophagus "primary gastrostomy and timely dilatation of the contracting scar will most probably prevent conditions which at present generally confront the surgeon in this class of cases, and are sometimes incurable."

